# Supplementary material for: Spatio-temporal evolution of habitat quality and its influencing factors in karst areas based on the InVEST model
Source: PLoS One. 2025 Mar 13;20(3):e0314161. doi: 10.1371/journal.pone.0314161 (PMC11906070; doi:10.1371/journal.pone.0314161)
Supplement: S1 Table — (DOCX) [file pone.0314161.s001.docx]

**S1 Table. Landscape index and its ecological significance**

| **Landscape index** | **Ecological significance** |
| --- | --- |
| NP | As the total number of landscape patches increases, landscape heterogeneity and fragmen. |
| AREA_MN | The larger the total area/number of patches the lower the landscape fragmentation. |
| LSI | Reflects the complexity of the shape of each patch, with larger values indicating more regular patches and a higher degree of agglomeration. |
| COHESION | Characterizing the natural connectivity of patches of the same type, the higher the value, the higher the clustering degree of patches. |
| CONTAG | It characterizes the degree of spread of landscape types - the higher the value, the more connected patches there are in the landscape. |
| SHDI | Reflect the richness of the landscape, describing the uniformity of the area size of different types of landscapes, and the larger the value, the more balanced the distribution of different patches in the landscape. |
| FN | Characterize the degree of landscape fragmentation (complexity of spatial structure), which may reflect the degree of human intervention in the landscape. |

* Note (NP: Number of Patches; AREA_MN: Mean of Patch Area; LSI: Landscape Shape Index; COHESION: Cohesion Index; CONTAG: Contagion Index; SHDI: Shannon’s Diversity Index; FN: Fragmentation Index)
