# Supplementary material for: Spatio-temporal evolution of habitat quality and its influencing factors in karst areas based on the InVEST model
Source: PLoS One. 2025 Mar 13;20(3):e0314161. doi: 10.1371/journal.pone.0314161 (PMC11906070; doi:10.1371/journal.pone.0314161)
Supplement: S2 Table — (DOCX) [file pone.0314161.s002.docx]

**S2 Table. Habitat threat factors**

| **Threat factors** | **Maximum impact distance/km** | **Weights** | **Recession type** |
| --- | --- | --- | --- |
| Town land | 10 | 1.0 | Index recession |
| Rural settlements | 8 | 0.8 | Index recession |
| Other building land | 9 | 0.9 | Index recession |
| Cropland | 6 | 0.6 | Linear recession |
| Unused land | 4 | 0.4 | Linear recession |
