# Supplementary material for: Spatio-temporal evolution of habitat quality and its influencing factors in karst areas based on the InVEST model
Source: PLoS One. 2025 Mar 13;20(3):e0314161. doi: 10.1371/journal.pone.0314161 (PMC11906070; doi:10.1371/journal.pone.0314161)
Supplement: S3 Table — (DOCX) [file pone.0314161.s003.docx]

**S3 Table. Habitat suitability and threat factors sensitivity of the landscape types in the Guizhou province**

| **Land use type** | **Habitat suitability** | **Town land** | **Rural settlements** | **Other building land** | **Cropland** | **Unused land** |
| --- | --- | --- | --- | --- | --- | --- |
| Cropland | 0.3 | 0.8 | 0.6 | 0.7 | 0 | 0.4 |
| Forest | 1.0 | 0.8 | 0.7 | 0.7 | 0.6 | 0.2 |
| Grassland | 1.0 | 0.7 | 0.5 | 0.6 | 0.5 | 0.6 |
| Wetland | 0.9 | 0.7 | 0.6 | 0.7 | 0.4 | 0.4 |
| Building | 0 | 0 | 0 | 0 | 0 | 0 |
| Unused land | 0.6 | 0.6 | 0.5 | 0.6 | 0.4 | 0 |
