# Supplementary material for: Spatio-temporal evolution of habitat quality and its influencing factors in karst areas based on the InVEST model
Source: PLoS One. 2025 Mar 13;20(3):e0314161. doi: 10.1371/journal.pone.0314161 (PMC11906070; doi:10.1371/journal.pone.0314161)
Supplement: S4 Table — (DOCX) [file pone.0314161.s004.docx]

**S4 Table. Result types of two-factor interaction**

| **Basis of judgment** | **Interaction type** |
| --- | --- |
| q(X1∩X2)＜Min[q(X1),q(X2)] | Non-linearity attenuation |
| Min[q(X1),q(X2)]＜q(X1∩X2)＜MaX[q(X1),q(X2)] | The single-factor nonlinearity decreases |
| q(X1∩X2)＞MaX[q(X1),q(X2)] | Two-factor enhancement |
| q(X1∩X2)=q(X1)＋q(X2) | independent |
| q(X1∩X2)＞q(X1)＋q(X2) | Nonlinear enhancement |
