# Supplementary material for: Spatio-temporal evolution of habitat quality and its influencing factors in karst areas based on the InVEST model
Source: PLoS One. 2025 Mar 13;20(3):e0314161. doi: 10.1371/journal.pone.0314161 (PMC11906070; doi:10.1371/journal.pone.0314161)
Supplement: Attachment — Submitted filename: Response to Reviewers 1,2,3.docx [file pone.0314161.s007.docx]

Dear Editors，

Greetings! We have made all the necessary changes to the article and have responded to each reviewer individually, addressing their questions in the order of questions 1, 2, and 3. If you have any further inquiries, please do not hesitate to contact us. We look forward to hearing from you.

Yours sincerely,

Chao Ma

Response to Reviewer 1 Comments

Dear Reviewers,

Thank you for recognizing our work. We will carefully review and correct the full text. Thank you for your hard work on this article.

Response to Reviewer 2 Comments

Dear Reviewers,

Thank you very much for your comprehensive and constructive comments on our article, which were very helpful for us to improve this manuscript. The details of the issues are as follows：

**Point 1:** Some of the language in the article needs further revision and polishing. I did not check all the references, and I noticed that some of them were not appropriate.

**Response 1:** Thank you for this comment. We attend English professionals to revise and embellish the language throughout the article. We checked references throughout and corrected inappropriate references. Detailed modifications are as follows: (Lines 495-661, Page 15-19):

2 Williams SE, Hobday AJ, Falconi L, Hero J, Holbrook NJ, Capon S, Bond NR, Ling SD, Hughes L. Research priorities for natural ecosystems in a changing global climate. Glob. Change. Bio. 2020; 26, 410-416.

7 Chen CZ, Ge XG, Sun L, Shao D, Ke WL. 9.Dramatic species loss · severe ecological overload · crossing the “earth boundary” · regional equity imbalances · “living on one planet” — interpreting *The Earth Vitality Report 2014*. Acta Ecologica Sinica, 2016; 36, 2779-2785.

8 Williams BA, Watson JEM, Butchart SHM, et al. A robust goal is needed for species in the Post‐2020 Global Biodiversity Framework. Conservation Letters, 2021; 14, e12778. <https://doi.org/10.1111/conl.12778.>

27 Han HQ, Liu Y, Gao HJ, Zhang YJ, Wang Z, Chen XQ, Tradeoffs and synergies between ecosystem services: A comparison of the karst and non-karst area. J Mt. Sci. 2020; 17, 1221–1234. https://doi.org/10.1007/s11629-019-5667-5.

59 IPBES W. Intergovernmental science-policy platform on biodiversity and ecosystem services. Summary for Policy Makers of the Global Assessment Report on Biodiversity and Ecosystem Services of the Intergovernmental Science-Policy Platform on Biodiversity and Ecosystem Services. IPBES Secretariat, Bonn, Germany, 2019.

Response to Reviewer 3 Comments

Dear Reviewers,

Thank you very much for your comprehensive and constructive comments on our article, which were very helpful for us to improve this manuscript. The details of the issues are as follows：

**Point 1:** For Table 1, make sure the font is New Roman and not a mix.

**Response 1:** Thank you for this comment. We have made changes based on the suggestions. We have changed the fonts in Table 1 uniformly to the Times New Roman font. Detailed modifications are as follows: (Lines 122, Page 4):

**Table 1.** Data source

| **Name** | **Type** | **Data source** | **Note** | **Time** |
| --- | --- | --- | --- | --- |
| **Land use/Land cover** | Raster | Resource and Environment Science and Data Center  http://www.resdc.cn | 30m×30m | Accessed on 10 October 2023 |
| **Annual temperature** | Raster | China Meteorological Data Center, http://data.cma.cn/ | 1000m×  1000m |  |
| **Annual precipitation** | Raster | China Meteorological Data Center, http://data.cma.cn/ | 1000m×  1000m |  |
| **NDVI** | Raster | Resource and Environment Science and Data Center  http://www.resdc.cn | 1000m×  1000m |  |
| **GDP** | Raster | Resource and Environment Science and Data Center  http://www.resdc.cn | 1000m×  1000m |  |
| **Digital Elevation Model (DEM)** | Raster | Geospatial Data Cloud,  http://www.gscloud.cn | 30m×30m |  |
| **Population Density** | Raster | Resource and Environment Science and Data Center  http://www.resdc.cn | 1000m×  1000m |  |

**Point 2:** Row 143, needs to be written top space (no indentation).

**Response 2:** We have changed line 143 to be written top space. We have checked the entire article and corrected every error. Detailed modifications are as follows:

(Lines 129, Page 5): “where, *N* is the unit of variation.”

(Lines 143, Page 5): “where, L was the land use intensity index for ...”.

(Lines 151, Page 6): “where, $D_{\mathrm{xj}}$was the degree of habitat degradation.”

(Lines 158, Page 6): “where, $Q_{xi}$ is the habitat quality index..”.

(Lines 171, Page 6): “where, *I* was Moran's index.”

(Lines 180, Page 6): “where, the value of *Q* was the explanatory power...”.

**Point 3:** Table 3 needs to be revised and the land use transfer matrix can be referenced in the relevant literature.

**Response 3:** Thank you for this comment. We have made an addition to Table 3 which does contain. I apologize that this was an oversight in our work. Detailed modifications are as follows: (Lines 230, Page 8):

| **1990** | **2020** | | | | | | |
| --- | --- | --- | --- | --- | --- | --- | --- |
|  | **Cropland** | **Forest** | **Grassland** | **Waters** | **Building** | **Unused**  **land** | **Amount of change** |
| **Cropland** | 43591.08 | 3133.39 | 1193.72 | 254.81 | 1121.03 | 0.76 | 5703.71 |
| **Forest** | 3137.96 | 86077.76 | 4599.17 | 409.24 | 412.35 | 2.51 | 8561.23 |
| **Grassland** | 1515.03 | 3945.24 | 25200.50 | 157.01 | 372.51 | 1.24 | 5991.03 |
| **Waters** | 13.35 | 16.08 | 7.20 | 343.80 | 1.62 | 0.03 | 38.28 |
| **Building** | 17.66 | 11.12 | 7.30 | 3.72 | 501.80 | 0.01 | 39.81 |
| **Unused land** | 1.64 | 8.82 | 2.60 | 0.10 | 1.61 | 25.60 | 14.77 |
| **Amount of change** | 4685.64 | 7114.65 | 5809.99 | 824.88 | 1909.12 | 4.55 | 20348.83 |

We have modified Table 4 by referring to Liu et al [1].

[1] Liu JY, Ning J, Kuang WH, et al. Spatio-temporal patterns and characteristics of land-use change in China during 2010-2015. Acta Geographica Sinica, 2018, 73 (05): 789-802.

**Point 4:** Table 5, and also Table 6, the form of the table is a bit confusing.

**Response 4:** Thank you for this comment. We have modified Tables 5 and 6. Detailed modifications are as follows: (Lines 278, Page 10; Lines 324, Page 11):

**Table 5. Proportion and mean of each class of habitat quality**

| **Class** | **Classification range** | **Area percentage (%)** | | | |
| --- | --- | --- | --- | --- | --- |
|  |  | **1990** | **2000** | **2010** | **2020** |
| **I** | 0-0.2 | 0.34 | 0.38 | 0.62 | 1.60 |
| **II** | 0.2-0.4 | 27.97 | 28.10 | 27.95 | 27.19 |
| **III** | 0.4-0.6 | 0.12 | 0.13 | 0.28 | 0.65 |
| **IV** | 0.6-0.8 | 0.83 | 1.02 | 1.87 | 4.04 |
| **V** | 0.8-1 | 70.74 | 70.19 | 69.29 | 66.35 |

We have changed one of the expressions in Table 6. We use a line graph to express it, which is more intuitive and clearer.

**Fig 8. Detection results of driving factors in karst areas**
